# Metabolomics reveals novel blood plasma biomarkers associated to the BRCA1-mutated phenotype of human breast cancer

**DOI:** 10.1038/s41598-017-17897-8

**Published:** 2017-12-19

**Authors:** Bàrbara Roig, Marta Rodríguez-Balada, Sara Samino, Eric W.-F. Lam, Sandra Guaita-Esteruelas, Ana R. Gomes, Xavier Correig, Joan Borràs, Oscar Yanes, Josep Gumà

**Affiliations:** 1 Institut d’Oncologia de la Catalunya Sud (IOCS), Hospital Universitari Sant Joan de Reus, IISPV, Universitat Rovira i Virgili, Av. del Dr. Josep Laporte, 43204 Reus, Spain; 2 Metabolomics Platform, Department of Electronic Engineering (DEEEA), Universitat Rovira i Virgili, 43003 Tarragona, Catalonia Spain; 3 Biomedical Research Centre in Diabetes and Associated Metabolic Disorders (CIBERDEM), 28029 Madrid, Spain; 4 Department of Surgery and Cancer, Imperial Centre for Translational and Experimental Medicine (ICTEM), Imperial College London, Hammersmith Hospital Campus, London, W12 0NN UK

## Abstract

Hereditary breast and ovarian cancer syndrome (HBOC) is partly due to the presence of mutations in the *BRCA* genes. Triple-negative (TN) breast cancer (BC) shares histological characteristics with germline *BRCA1* mutation-associated tumours. We have investigated the metabolic profiles of human breast cancer (BC) cell lines carrying *BRCA1* pathogenic mutations by non-targeted liquid chromatography coupled to mass spectrometry technology. Based on our *in vitro* results, we performed a targeted metabolomic analysis of plasma samples from TN HBOC patients taking into account their *BRCA1* genotype. *BRCA1* promoter hypermethylation and the BRCAness phenotype of BC cell lines were also studied. The purpose of this study was to determine the metabolic signature of HBOC syndrome and TNBC patients and to evaluate the potential contribution of the metabolites identified to the genetic diagnosis of breast cancer. The present results show the existence of a differential metabolic signature for BC cells based on the BRCA1 functionality. None of the studied BC cell lines presented hypermethylation of the *BRCA1* promoter region. We provide evidence of the existence of free methylated nucleotides capable of distinguishing plasma samples from HBOC patients as *BRCA1*-mutated and *BRCA1* non-mutated, suggesting that they might be considered as BRCA1-like biomarkers for TNBC and HBOC syndrome.

## Introduction

Pathogenic germline mutations in the *BRCA1* and *BRCA2* genes predispose patients to hereditary breast and ovarian cancer syndrome (HBOC)^1^. Approximately 15–20% of HBOC cases are associated with the presence of germline mutations in *BRCA1* or *BRCA2*. Although the frequency of mutations in these genes is very low in the general population (<0.005%), when they occur, the risk of cancer is very high (relative risk 10). However, it is important to note that the risk associated with these mutations is only approximate because there are other genetic and environmental factors that can modify the penetrance and phenotypic expression of these mutations^1^.

BRCA1 and BRCA2 are classic tumour suppressor proteins that are involved in the DNA homologous recombination repair (HRR) pathway, which is activated in response to DNA double-strand breaks (DSBs)^2^.

Tumours that arise in patients carrying *BRCA1* germline mutations tend to be triple negative breast cancers (TNBC). TNBC is a heterogeneous group of tumours characterized by the absence of expression of histopathological markers such as the oestrogen receptor (ER), progesterone receptor (PgR) and ERBB2/HER2 oncogene. TNBC is biologically and clinically more aggressive than receptor- or oncogene-expressing tumours. These tumours frequently arise in younger patients, are generally larger in size at diagnosis, have a high histological grade and are responsible for many BC-related deaths^3–6^.

Otherwise, the presence of *BRCA1* somatic mutations in sporadic TNBC is rare, accounting for less than 15% of cases^7^. Therefore, other somatic events that inactivate BRCA1 can predispose to sporadic breast tumours. In fact, approximately 10–30% of sporadic cases of BC show somatic *BRCA1* promoter hypermethylation, which silences its expression^8–10^. Recently, TNBC specimens were found to frequently present a BRCA1-like profile based on comparative genomic hybridization-specific array analysis (aCGH), revealing phenotypic characteristics that are similar to those of hereditary breast tumours from carriers of germline *BRCA1* mutations. This BRCA1-like profile is also referred to as “BRCAness”^11^. BRCAness has been defined as a phenocopy of *BRCA1* or *BRCA2* pathogenic mutations, with the existence of an HRR tumour defect in the absence of a *BRCA1* or *BRCA2* germline mutation^12^. Defects in other genes that modulate the HRR process may also cause a BRCAness phenotype.

Metabolomics is the most recently developed of the omics sciences and refers to the study, identification and quantification of metabolites. Metabolites are small molecules (<1500 Da) that are the product of metabolic reactions or the consequences of the activity of genes and proteins. The presence or absence of certain metabolites and the changes in their concentrations may be indicative of a particular disease or predisposing factors^13^. To perform metabolic characterization, metabolomics uses analytical complementary techniques such as mass spectrometry (MS) or nuclear magnetic resonance (NMR). Metabolomic measurements can provide untargeted and semi-quantitative measurement of metabolites in different matrices, such as cell lines^14^ and blood serum or plasma^15^. Once the measurements are acquired, accumulated data are obtained, and statistical techniques are used to identify relevant biochemical information from these data. Thus, the metabolic profile of a particular disease can be determined^13,14^. This makes it possible to better characterize the phenotype of a disease, and consequently, the phenotypic expression of genetic mutations.

In recent years, an increasing number of metabolomic studies aimed at biomarker discovery for different diseases, including breast cancer, have been reported^15–19^. Metabolomics is now considered a powerful technology for the identification of biomarkers and altered metabolic pathways in cancer^20,21^, which include glycolysis, amino acid metabolism, nucleotide synthesis, phospholipid and fatty acid metabolism^19,20,22,23^.

One effective source for novel cancer biomarkers that has attracted less attention is the conditioned media of cultured cancer cell (secretome)^24^. The secretome contains organic molecules released by tumour cells into the extracellular space that could better reflect the activity of cancer in human body fluids such as blood plasma or serum^25,26^.

The purpose of the present project was to analyse and to characterize the metabolic profile of human breast cancer cell lines and plasma samples of TNBC from HBOC patients who were carriers and non‐carriers of germline mutations in the *BRCA1* gene. Alterations in the concentrations of certain metabolites detected in the secretome of these cell profiles can be candidates as future biomarkers of HBOC syndrome. Likewise, alterations in the concentrations of metabolites in cell pellets could provide new insights into the pathological heterogeneity of TN breast tumours and its relationship with BRCA1.

## Results

### BC cell lines segregate into three different metabolic clusters based on their *BRCA1* genotype

**Figure 1:**
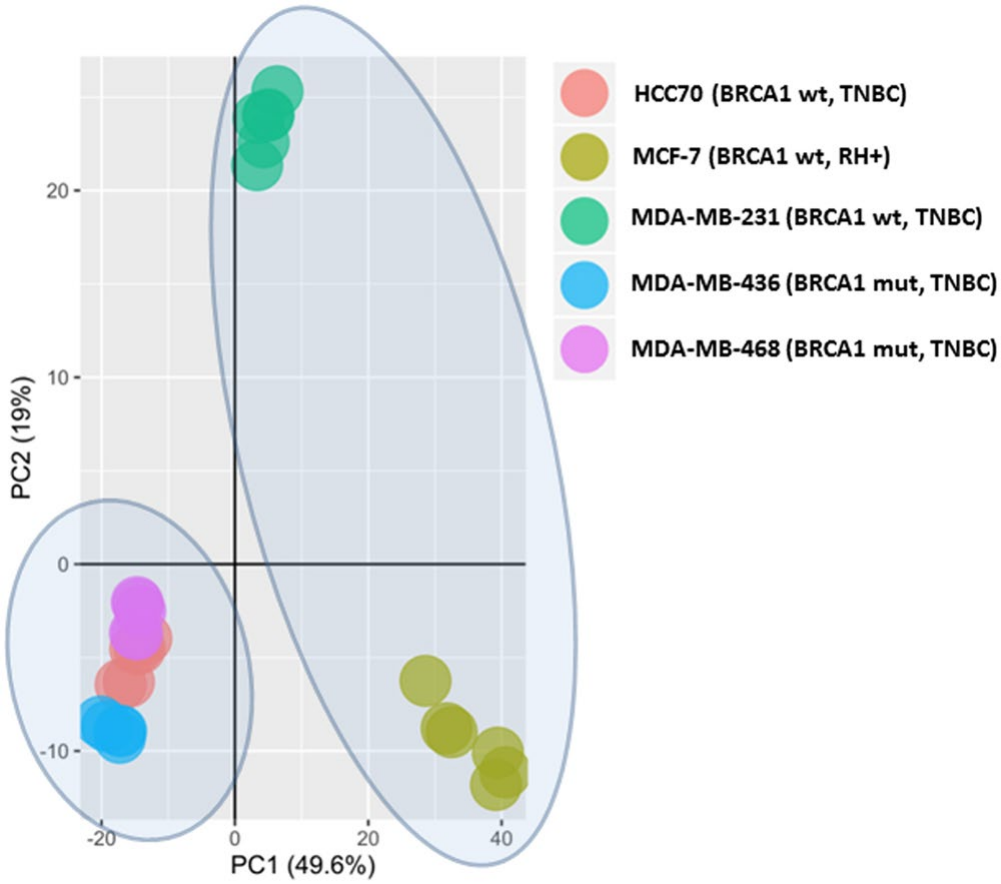
The results of the principal component analysis (PCA) score plots of the breast cancer cell lines analysed by LC-MS. Scrutiny of the analysed breast cancer cell lines indicated the similarities and differences between BRCA1 genotypes.

An unsupervised principal component analysis (PCA) was carried out to reveal the global metabolic differences in BC cell lines according to their *BRCA1* genotype. The PCA score plot showed clear separations among the BC cell lines that were analysed. As shown in Fig. 1, we identified different clusters based on the metabolic signature of the cell lines according to their *BRCA1* genotype. The first principal component (PC1) accounted for the greatest variability in the data set (49.6%) and revealed a clear homogeneous metabolic cluster composed of the MDA-MB-436, MDA-MD-468 and HCC70 BC cell lines, which differed from that of the MDA-MD-231 and MCF7 cell lines. Notably, although the HCC70 BC is a non-mutated *BRCA1* BC cell line, it showed a metabolic profile that clustered with the BC cell lines that are carriers of pathogenic *BRCA1* mutations (Fig. 1).

### Promoter methylation of the *BRCA1* gene

Considering that, at the metabolic level, the HCC70 cell line showed a phenotype similar to *BRCA1*-mutated BC cell lines, we examined whether its BRCA1-like metabolic phenotype was caused by *BRCA1* promoter methylation. We found that the HCC70 cell line was not hypermethylated in the *BRCA1* promoter region (Supplementary Table S1). None of the other BC cell lines that were analysed showed aberrant methylation of the *BRCA1* promoter region (Supplementary Table S1).

### BRCAness phenotype in BC cell lines

A commercial MLPA assay was used to test the BRCA1-like genomic profile of BC cell lines. This profile represents the BRCAness and can be used as a marker of defects in the HRR pathway. The BRCAness phenotype is related to sporadic TNBC, which shares clinical and histological characteristics with germline *BRCA1-*mutated breast tumours (young age, high grade and negativity for the ER, PgR and HER2). Based on the clinicopathological features of the HCC70 cell line (high-grade TNBC from a 45-year-old patient, no *BRCA1* mutation) and according to BRCA1 protein expression previously reported in the same cell lines^27^, we hypothesized that the BRCA1-like metabolic profile of the HCC70 cell line can be explained by BRCAness. Our results show that the HCC70 BC cell line presented a BRCAness profile (Table 1). Similarly, we found that the mutated *BRCA1* cell line, MDA-MB-468, also showed a BRCAness profile (Table 1). Both cell lines were derived from a basal BC subtype and showed negativity for expression of the ER, PgR and HER2. The other BC cell lines that were analysed did not show a BRCAness phenotype (sporadic-like breast tumours) (Table 1). Notably, the MDA-MB-436 BC cell line showed a sporadic-like phenotype (Table 1). MDA-MB-436 is a TNBC cell line that is a carrier of a splice-site mutation located in intron 20 of the *BRCA1* gene^28^. As described, *BRCA1* mutation prediction by BRCAness shows a sensitivity of only 83%^29,30^. This MLPA BRCA1ness probemix is designed to detect amplifications and deletions of one or more sequences in selected genes and chromosomal regions that were previously reported to be altered in breast cancer and only heterozygous deletions of the recognition sequences gives a BRCA1ness profile^29,30^. Specifically, the probes for the BRCA1 gene are located in intron 19 and exon 2. One possible explanation of the non-BRCA1ness profile detected in the mutated MDA-MB-436 cell line is that the single nucleotide mutation is located outside the BRCA1 region probe. In addition, MDA-MB-436 can be carrier of a heterozygous deletion outside of the region selected in the probemix so it can not be detected.

**Table 1:** MLPA BRCAness phenotype of breast cancer cell lines analysed.

Breast cancer cell line	Phenotype	
	BRCA1_like	Sporadic_like
MCF7	NO (0.02664)	YES (0.97336)
MDA-MB-231	NO (0.01184)	YES (0.98816)
MDA-MB-436	NO (0.00125)	YES (0.99875)
MDA-MB-468	YES (0.97079)	NO (0.02921)
HCC70	YES (0.99405)	NO (0.00595)

The cut-off value to classify a cell line as BRCA1-like was set at 0.5. Below this value, the cell line was classified as sporadic-like.

### Identification of metabolites that define a BRCA1-like BC phenotype

**Figure 2:**
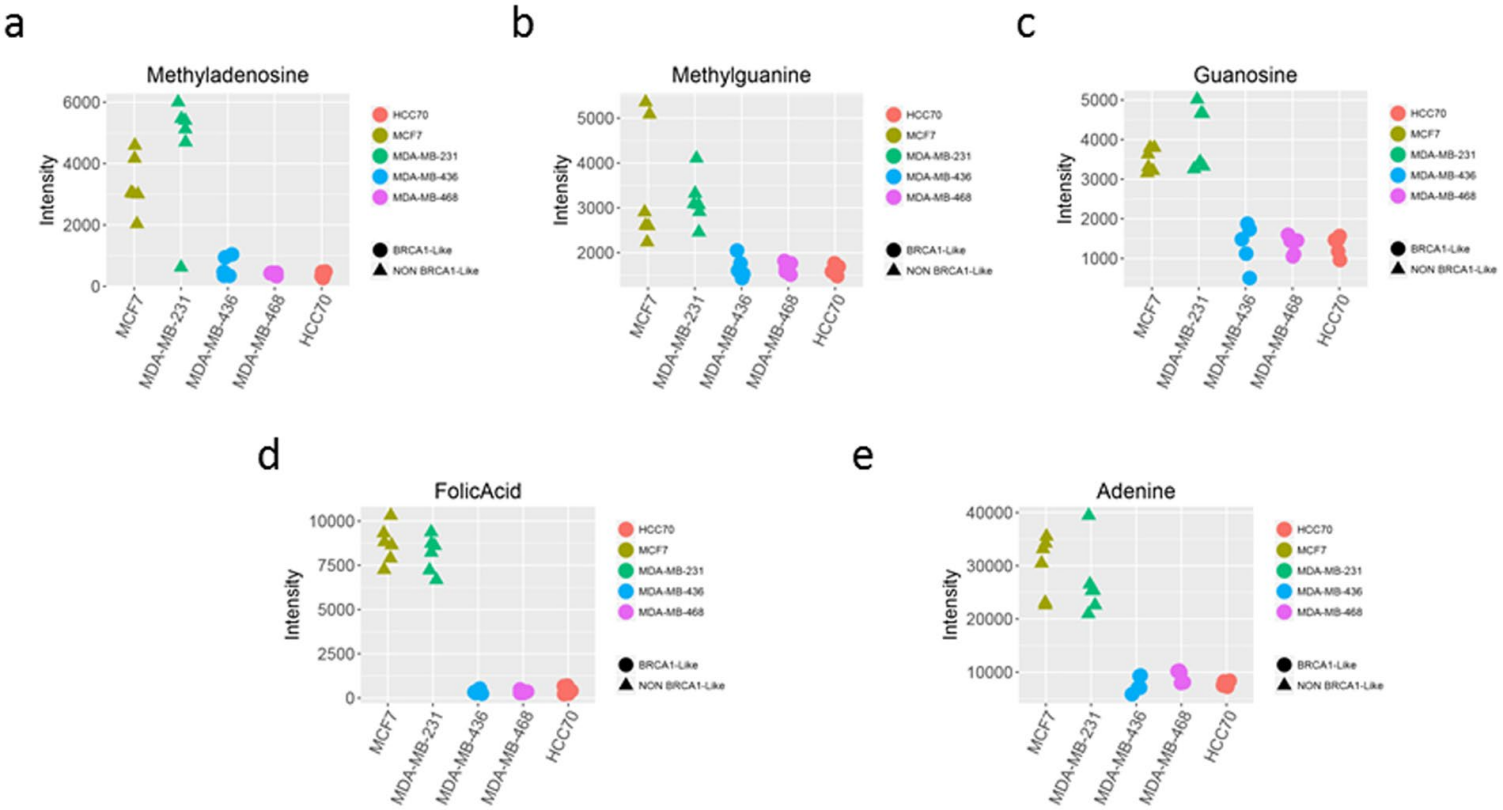
Absolute quantification of the expression levels of the identified metabolites that might be used to define a BRCA1-like breast cancer phenotype. All of these metabolites were down-regulated in cell lines that showed a BRCA1-like metabolic profile compared to the cell lines that showed a non-BRCA1-like phenotype.

According to the classification of maximum variability (based on PC1 from Fig. 1), we performed univariate statistical analysis considering MCF-7 and MDA-MB-231 as non-BRCA1-like cell lines and HCC70, MDA-MB-436 and MDA-MB-468 as BRCA1-like cell lines. From our non-targeted LC-MS metabolomic study, we identified by exact mass and MS/MS nine metabolites whose distribution was significantly different between the clusters: adenine, arginine-aspartate, folic acid, guanosine, methyladenosine, methylguanine, phenylacetylglycine, thiamine pyrophosphate and uridine (Table 2). All metabolites were significantly under-represented in BRCA1-like BC cell lines compared with *BRCA1* wild-type (wt) BC cell lines (Fig. 2).

**Table 2:** Differential significant metabolites identified in the cells and secretome of the breast cancer cell lines to discriminate BRCA1-like and non BRCA1-like breast cancer profile.

Metabolite	m/z	RT (sec)	Cell Pellet		Secretome		Formula
			P. adj value	FC	P. adj value	FC	
Adenine	136.0612	263.7	3.4E-03	−2.61	1.2E-01	3.10	C_10_H_13_N_5_O_4_
Arginine-Aspartate	290.1439	65.1	2.9E-03	−3.71	ND	ND	C_10_H_19_N_5_O_5_
Folic Acid	442.1441	295.5	8.8E-04	−17.4	3.6E-14	−3.6	C_19_H_19_N_7_O_6_
Guanosine	284.0804	243.3	2.4E-03	−2.28	ND	ND	C_10_H_13_N_5_O_5_
Methyladenosine	282.1178	261.1	8.4E-03	−5.52	8.4E-04	−3.6	C_11_H_15_N_5_O_4_
Methylguanine	166.0712	222.5	1.7E-02	−1.68	1.5E-05	−3.7	C_6_H_7_N_5_O
Phenylacetylglycine	194.0801	348.6	3.2E-04	−5.09	8.8E-10	−2.3	C_10_H_11_NO_3_
Thiamine pyrophosphate	425.0413	65.8	5.8E-03	−2.64	2.7E-16	−2.9	C_12_H_18_N_4_O_7_P_2_S
Uridine	243.0636	190.6	2.4E-03	−4.29	ND	ND	C_9_H_12_N_2_O_6_

m/z: mass to charge ratio; RT: retention time; FC: fold change expressed as log2; ND: not detected.

On the other hand, when we analysed the secretome of the BC cell lines, we found that six of the nine metabolites identified in cell pellets were also secreted (Table 2). We found statistically significant differences in all of these secreted metabolites between the *BRCA1* mutated and non-mutated BC cell lines, except for the adenine metabolite (Table 2).

### Metabolite validation in plasma samples from TN HBOC patients

The discriminatory power of the metabolic BRCA1-like phenotype signature was tested in 35 human plasma samples obtained from TN HBOC patients who were carriers and non-carriers of *BRCA1* mutations.

Of the metabolites identified in the secretome of BC cell lines, two metabolites were not detected in all the human plasma samples analysed (Table 3).

**Table 3:** Candidate metabolites analysed to discriminate BRCA1-like breast cancer profile in plasma samples of HBOC patients.

Metabolite	TN HBOC BRCA1 mutated		TN HBOC BRCA1 wt		P. adj value
	Mean	SD	Mean	SD	
Adenine	0.32	0.19	0.56	0.42	**0**.033*
Arginine-Aspartate	ND	ND	ND	ND	ND
Folic Acid	0.04	0.05	0.02	0.02	0.41
Guanosine	0.51	0.21	0.58	0.78	0.10
1-Methyladenosine	34.18	7.81	35.35	9.24	0.76
N6-Methyladenosine	0.42	0.35	0.78	0.42	**0**.012*
1-Methylguanine	0.56	0.29	0.76	0.33	**0**.033*
7-Methylguanine	9.32	1.85	9.15	2.14	0.55
Phenylacetylglycine	1.60	2.55	2.47	2.64	0.18
Thiamine pyrophosphate	ND	ND	ND	ND	ND
Uridine	680.67	262.85	614.48	273.78	0.41

TN: triple negative; HBOC: hereditary breast and ovarian cancer; wt: wild-type; SD: standard deviation; ND: not detected; * Statistically significant (p value < 0.05).

**Figure 3:**
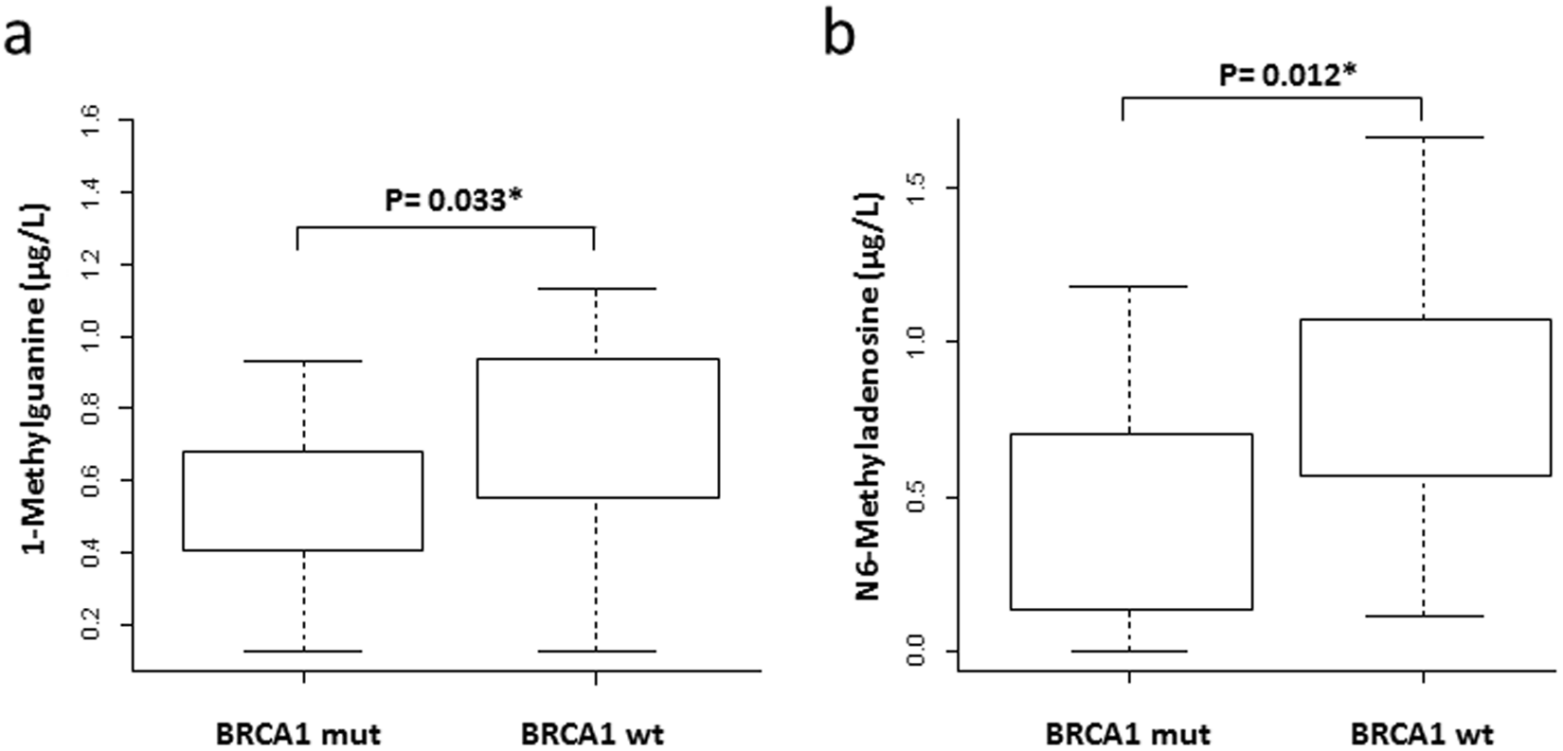
A boxplot representation of the absolute concentrations of N6-methyladenosine and 1-methylguanine metabolites in human plasma samples. Both are methylated nucleotides that were differentially expressed between TNBC samples according to their BRCA1 genotype.

With the application of the Kolmogorov test, we found that the levels of adenine, N6-methyladenosine and 1-methylguanine detected in the plasma samples of patients who were carriers of *BRCA1* mutations were significantly lower than those in patients who were not carriers of *BRCA1* mutations (Fig. 3). Interestingly, N6-methyladenosine and 1-methylguanine are metabolites directly related to well-known BC events, such as methylation processes^31–34^. The other secreted metabolites identified in BC cell lines did not show any statistically significant differences when we compared both groups of HBOC plasma samples analysed (Table 3).

## Discussion

Metabolomics is a useful tool for the identification of biomarkers and metabolic disturbances that are present in cancer. Since cancer is a disease with metabolic alterations, there has been an increasing number of published studies related to BC and metabolomics^20,35^. In this study, we presented the metabolomic based profile of BC cell lines for the characterization and identification of the breast cancer phenotype linked to the *BRCA1* genotype. For this purpose, we used several human BC cell lines characterized by the presence of pathogenic mutations in the *BRCA1* gene, and we compared them with BC cell lines without *BRCA* mutations. Then, we tested the discriminatory power of the identified metabolites in a sample cohort of plasma samples from patients who were or were not carriers of *BRCA1* mutations. We evaluated the contributions of these metabolites to the genetic diagnosis of HBOC syndrome.

Our results revealed phenotypic similarities based on the metabolic data that make it possible to distinguish between cell lines depending on the presence of alterations linked to *BRCA1*. We also provide evidence of common metabolic features that were identified despite the strong heterogeneity observed among the BC cell lines.

None of the BC cell lines with a *BRCA1* mutation showed *BRCA1* promoter methylation, supporting the hypothesis that genetic and epigenetic inactivation of BRCA1 is mutually exclusive^29^.

We herein provide the first description of the BRCAness phenotype of the HCC70 TNBC cell line and report that the BRCAness profile shares common metabolic features with BRCA1-mutated tumours. Consistent with previous studies stating that BRCAness phenotypes of TNBC were histologically and clinically similar to those of hereditary BC tumours from patients who were carriers of pathogenic BRCA1 mutations, our results show a similar observation at metabolic level, evidencing that BRCA1-mutated cell lines and genomic BRCAness (and non-BRCA1 mutated) HCC70 cell line are metabolically similar compared to non-BRCA1 mutated and non-BRCAness BC cell lines^29,30^.

There is an urgent need to search for and identify new clinical biomarkers of cancer. The ideal biomarker is one that can be detected easily and non-invasively and is capable of discriminating patient subgroups. Most of the biomarkers routinely used in the clinical setting are secreted and expressed in biological fluids such as blood. In this context, analysing the secretome from the conditioned medium of cultured tumour cells could be a way to discover new tumour-specific biomarkers^25,26,36^. In this study, we identified alterations in the concentrations of nine metabolites in the metabolic profiles of cell pellets, six of which were also differentially regulated in the cells’ secretomes. Thus, these metabolites may represent potential BC biomarkers linked to BRCA1. A quantitative validation of these secreted metabolites in human samples showed that the plasma levels of adenine, N6-methyladenosine and 1-methylguanine could distinguish TN HBOC patients who were carriers of pathogenic *BRCA1* mutations from TN HBOC patients who were non-carriers of BRCA mutations, supporting their putative role as BRCA1-like biomarkers in patients with HBOC syndrome and TNBC.

Adenine is a compound necessary for nucleotide synthesis and is considered as an indicator of abnormal cancer cell proliferation^37^. A correlation between an increase of adenine and guanine and cancer cell metastasis due to imbalance of enzymatic activity of purine metabolism has been described^38^. Moreover, a recent metabolomic profile of human breast cancer cell lines has identified adenine as a putative prognostic biomarker of breast cancer metastasis^39^.

1-Methylguanine is a methylated-derived purine that was originally found to be elevated in urinary and serum samples of breast cancer patients, and it was suspected to alter DNA methylation processes^40^. Recently, 1-methylguanine has been described as one of the N-methyl adducts occurring at the DNA, which are repaired by the adaptive response of AlkB enzyme in order to avoid adverse effect of these DNA lessions^31^.

N6-Methyladenosine is the most frequent methylated nucleoside on RNA in eukaryotes with epitranscriptomic regulation functions^32–34^. It has been described that 6-methyladenosine RNA editing is linked to breast cancer transformation, and it is suspected to be a DNA methylation marker in eukaryotes involved in transcription regulation^41,42^.

It is well established that patients with BC present disturbances in gene methylation processes that can silence gene expression. Epigenetic events in conjunction with genetic alterations are important in BC development, and multiple studies have used BRCA1 as an example^43,44^. Hypermethylation of the BRCA1 promoter region and transcriptional repression of BRCA1 has been described in sporadic breast cancer^45–47^. These tumours are histologically and molecularly similar to BRCA1-associated breast tumours^48–50^. Recently, Stefansson *et al*. described the existence of different methylation signatures for BC tumour subtypes. These researchers showed a gene body hypomethylation signature that was exclusively associated with the basal-like subtype, a high-grade subgroup of tumours characterized by negativity for the ER, PgR and HER2 expression and related to a poor prognosis^43^. Hypomethylation of gene bodies has been described as a characteristic of repressed genes *in vitro* ^51^. In this context, an hypermethylation analysis of *BRCA1* promoter regions was negative for all of the BC cell lines examined in the present study. In addition, we found under-representation of methylated nucleotides in BC cell lines derived from tumours that were classified as TNBC, in addition to altered BRCA1 functionality. These findings were confirmed in plasma samples of TNBC from HBOC patients who were carriers and non-carriers of *BRCA1* mutations. Taken together, our results suggest that methylation events in DNA and/or RNA could be regulated by BRCA1.

Despite the high heterogeneity of BC as a disease, especially among cases of TNBC, we found a common BRCA1-like signature in BC cell lines according to the presence of defects in *BRCA1* functionality (i.e., caused by a *BRCA1* mutation or BRCAness phenotype). Validation of this metabolic BRCA1-like signature in TNBC is necessary to confirm whether it could facilitate the diagnosis and/or treatment of patients. Specifically, validation of this BRCA1-like metabolic profile in TNBC could aid in the prediction of resistance to taxanes and the sensitivity to platinum and PARP inhibitor agents. PARP1 is a critical enzyme for the base excision repair pathway, and its inhibition by RNA interference or chemical inhibitors leads to severe, highly selective toxicity in BRCA1- and BRCA2-defective cells. This toxicity results in chromosomal instability, cell cycle arrest and subsequent apoptosis, most likely due to the persistence of DNA lesions that are normally repaired by homologous recombination. Therefore, sensitivity to PARP inhibition depends on HRR deficiency^52–54^.

Further studies in a larger cohort of plasma patients and tumour specimens are needed to validate the present results. The implementation of these biomarkers may refine breast cancer diagnosis and perhaps enable personalized treatments.

## Methods

### BC cell lines

The BC cell lines analysed in the study were MCF-7, HCC70, MDA-MB-231, MDA-MB-436 and MDA-MB-468. The clinicopathological characteristics of these human BC cell lines are summarized in Supplementary Table S2. Briefly, all cell lines, except for MCF-7, are negative for the expression of hormone receptors, and are classified as basal-like BC. MCF-7, HCC70 and MDA-MB-231 cell lines are considered wild-type cells for *BRCA*, while the MDA-MB-436 and MDA-MB-468 cell lines are carriers of *BRCA1* pathogenic mutations^15^.

### BC patients

The study included 35 human plasma samples from TNBC patients who underwent BRCA genetic testing at the Genetic Counseling Unit of the Institute of Oncology of South Catalonia (IOCS). Of these, 23 TNBC patients were carriers of a *BRCA1* germline mutation and 12 patients were non-carriers of *BRCA1* or *BRCA2* germline mutations. Plasma was extracted by centrifuging whole blood at 3000 rpm for 10 min at room temperature. All extracted plasma samples were aliquoted and stored at −80 °C. We only used plasma samples that had not been previously thawed. Fresh tumour samples from patients were not available because the target of the BRCA genetic testing in hereditary breast cancer susceptibility is blood-DNA.

The study was approved by the Ethics Committee of Clinical Research of Sant Joan University Hospital, written informed consent was obtained from all participants and all experiments were performed in accordance with relevant guidelines and regulations.

### Cell cultures

MCF-7, HCC70, MDA-MB-231, MDA-MB-436 and MDA-MB-468 BC cells were obtained from the American Type Culture Collection (LGC Standards, Middlesex, UK) and were authenticated by Cancer Research UK (London, UK). MCF-7 and MDA-MB-468 cells were maintained in Dulbecco’s modified Eagle’s medium (DMEM) (Sigma-Aldrich, Madrid, Spain) supplemented with 10% foetal calf serum (FCS), 4 mM glutamine and 100 U/mL penicillin/streptomycin (Sigma-Aldrich, Madrid, Spain). HCC70 cells were cultured in RPMI 1640 (Gibco, Madrid, Spain) supplemented with 10% foetal calf serum. MDA-MB-231 cells were grown in phenol red-free DMEM (Invitrogen, Barcelona, Spain) supplemented with 10% foetal calf serum (FCS), 100 Units/mL penicillin, 100 Units/mL streptomycin and 2 mM L-glutamine. MDA-MB-436 cells were grown in Leibovitz’s L-15 medium (Gibco, Madrid, Spain) supplemented with 10% horse serum (Invitrogen, Barcelona, Spain). All cell lines were grown in an atmosphere of 10% CO_2_ at 37 °C except MDA-MB-436 cells, which were grown without CO_2_ at 37 °C.

### DNA isolation

Total DNA from human BC cell lines was extracted using a DNeasy Blood and Tissue kit (Qiagen, Madrid, Spain) according to the manufacturer’s instructions. DNA extraction was performed in duplicate for each human breast cell line culture.

### Untargeted LC-MS and MS/MS analysis of breast cancer cell lines

A volume of 220 µL of methanol, followed by 440 µL of dichloromethane, was added to each cell pellet. Samples were mixed vigorously for 30 sec and ultrasonicated on ice for 1 min. Then, 140 µL of Milli-Q water was added. Samples were mixed vigorously for 30 sec and were ultrasonicated on ice for 1 min. The samples were then incubated for 30 min on ice followed by centrifugation at 15,000 rpm (4 °C) for 15 min, and the aqueous phase was transferred to an LC-MS vial.

The untargeted metabolomics analysis on cell lines was performed using LC/ESI-QTOF. Samples were injected into an UHPLC system (1290 Agilent) coupled to a quadrupole time-of-flight (QTOF) mass spectrometer (6550 Agilent Technologies) operated in positive (ESI +) or negative (ESI−) electrospray ionization mode. Metabolites were separated using C18-RP (ACQUITY UPLC HSS T3 1.8 μm, Waters) for ESI + and C18-RP (ACQUITY UPLC BEH 1.7 μm) for ESI−. When the instrument was operated in positive ionization mode, the solvent system was A = 0.1% formic acid in water and B = 0.1% formic acid in acetonitrile. When the instrument was operated in negative ionization mode, the solvent system was A = 1 mM NH_4_F in water and B = acetonitrile^55^. The linear gradient elution started at 100% A (time 0–2 min) and finished at 100% B (10 min). The injection volume was 1 μL for cell pellets. The ESI conditions were as follows: gas temperature, 150 °C; drying gas, 11 L min^−1^; nebulizer, 30 psig; and fragmentor, 120 V. The instrument was set to acquire over the m/z range of 100–1200, with an acquisition rate of 3 spectra/sec. Samples were randomized to reduce systematic error associated with instrumental drift. MS/MS was performed in targeted mode, and the instrument was set to acquire over the m/z range of 45–1000, with an iso width (the width half-maximum of the quadrupole mass bandpass used during MS/MS precursor isolation) of 1.3 m/z. Samples were measured in triplicate at collision energies of 10, 20 and 40 V. All metabolites were identified in accordance with Level 1 or 2 of the Metabolomics Standards Initiative^56^, namely, by comparison with authentic chemical standards analysed in our laboratory (methylated nucleosides) or public/commercial spectral libraries, respectively. Quality control samples (QC) consisting of pooled samples of all samples were used. QC samples were injected before the first study samples and were then analysed periodically while analysing the study samples.

### BRCA1 promoter methylation

Hypermethylation of the BRCA1 promoter region was assessed using the commercial SALSA MS-MLPA probemix, ME001-C2 Tumour Suppressor-1, SALSA MS-MLPA probemix ME002-C1 Tumour Suppressor-2, and a new developed SALSA MS-MLPA probemix ME053-X1 BRCA1-BRCA2 according to the manufacturer’s protocol (MRC-Holland, The Netherlands). DNA fragments were analysed using an ABI 3500 Sequencer (Life Technologies, Spain). The results of multiplex-ligation-dependent probe amplification (MLPA) were normalized and analysed using the Coffalyser program as recommended (www.mlpa.com). Each BC cell line was analysed twice.

### BRCAness phenotyping

BRCAness profiling was performed using the commercial multiplex ligation-dependent probe amplification (MLPA) probemix P376-B2 BRCA1ness (MRC-Holland, The Netherlands). The MLPA results were normalized and analysed using the combination of the Coffalyser program and the Prediction Analysis for Microarrays (PAM) algorithm in R (www.r-project.com) as suggested by the supplier (www.mlpa.com). The cut-off value to classify a cell line as BRCAness was set at 0.5. Before we performed the cell line analysis, we performed the recommended training data set analysis. Each BC cell line was analysed twice.

### Targeted LC-MS/MS analysis of plasma samples from TN HBOC patients

Quantitative determination and validation of the identified cell lines’ metabolites were performed in 35 human plasma samples from TN HBOC patients by ultra-high-resolution liquid chromatography coupled to triple quadrupole mass spectrometry (UHPL-QqQ/MS).

For the chromatographic analysis, an ACQUITY UPLC HSS T3, 1.8 µm, 2.1 mm × 100 mm chromatographic column was used, which was maintained at 23 °C. Water was used with 0.1% HCOOCH as solvent A and acetonitrile was used as solvent B. The column flow was 0.40 mL/min and the mobile phase gradient conditions were as follows: 0–100% B (0–10 min), 100% B isocratic (10–13 min), 100–0% B (13–15 min). A 3 min post-run was applied. The injection volume was 5 µL. The ionization was carried out in an electrospray (ESI) source with positive polarity and applying a temperature and drying gas flow (N_2_) of 290 °C and 18 L/min respectively, a pressure of the nebulizer gas (N_2_) of 20 psi, and a temperature and sheath gas flow (N_2_) of 350° and 10 L/min, respectively. The voltage of the fragmentation was 380 V, the capillary was 3500 V and the nozzle voltage was 750 V. The detection was performed with a triple quadrupole detector (QqQ) by means of acquisition in Multiple Reaction Monitoring mode (MRM), applying a cell acceleration voltage of 5 V. To validate the quantitative method, we evaluated the following parameters: calibration lines, linearity, limits of detection and quantification (LoD and LoQ, respectively), accuracy and precision by analysing standard solutions and samples with standard solutions added to generate different concentrations.

### Data analysis and statistical methods

Untargeted LC-MS (ESI +and ESI− mode) data of BC cell lines were processed using the XCMS software (version 1.38.0) to detect and align features^57^. A feature was defined as a molecular entity with a unique m/z and a specific retention time. The XCMS analysis of these data provided a matrix containing the retention time, m/z value, and an integrated peak area of greater than 11,000 features after the analytical variability had been corrected^58^. Univariate and multivariate statistical analyses were performed using R Studio (version 1.3). For the univariate statistical analysis, a t-test was performed, and the p value was corrected using a false discovery rate. Differentially regulated metabolites (fold-change > 1.5) that passed our statistical criteria (adjusted p-value < 0.05) were characterized by LC-qTOF MS/MS and identified using the METLIN database or authentic chemical standards acquired in our laboratory with the same analytical method.

Statistical analyses of human plasma were performed using R (www.r-project.org). Continuous data were analysed with the Kolmogorov test (mean ± standard deviation). Statistical tests were two-sided and p-values < 0.05 were considered statistically significant.

## Electronic supplementary material

Supplemental File
