# Supplementary material for: Metabolomics reveals novel blood plasma biomarkers associated to the BRCA1-mutated phenotype of human breast cancer
Source: Sci Rep. 2017 Dec 19;7:17831. doi: 10.1038/s41598-017-17897-8 (PMC5736621; doi:10.1038/s41598-017-17897-8)
Supplement: Supplementary file 1 — Supplemental File [file 41598_2017_17897_MOESM1_ESM.pdf]

Metabolomics reveals novel blood plasma biomarkers associated to the BRCA1-mutated phenotype of human breast cancer

Barbara Roig[1+], Marta Rodríguez-Balada[1+], Sara Samino[2,3], Eric W.-F. Lam[4], Sandra Guaita-Esteruelas[1], Ana R. Gomes[4], Xavier Correig[2,3], Joan Borràs[1], Oscar Yanes[2,3], Josep Gumà[1]*

Supplementary Table 1. BRCA1 and BRCA2 promoter hypermethylation percentages of the breast cancer cell lines analysed by methylation MS-MLPA

Breast cancer	ME001-C2 assay		ME002-C1 assay		ME053-X1 assay						
**cell line**	**BRCA1**	**BRCA2**	**BRCA1**	**BRCA2**	**BRCA1.1**	**BRCA1.2**	**BRCA1.3**	**BRCA2.1**	**BRCA2.2**	**BRCA2.3**	**BRCA2.4**
MCF7	ND	ND	ND	ND	ND	ND	ND	ND	ND	ND	ND
MDA-MB-231	2%	0%	3%	0%	0%	2%	2%	4%	3%	0%	0%
MDA-MB-436	2%	0%	0%	0%	1%	2%	1%	6%	2%	0%	0%
MDA-MB-468	3%	0%	0%	0%	0%	2%	0%	4%	0%	0%	0%
HCC70	1%	0%	0%	6%	0%	3%	0%	4%	4%	1%	0%

ND: No DNA available

Supplemmentary Table 2. Clinicopathological characteristics of the human breast cancer cell lines analyzed

Cell Line Name	BRCA1 Genotype	TP53 Status	Immunoprofile	Subtype	Tumour type
**MCF-7**	WT	WT	ER+, PgR+/-, HER2-	Luminal	Metastasic adenocarcinoma
**HCC70**	WT	c.743G>A	ER-, PgR-, HER2-	Basal	Ductal carcinoma
**MDA-MB-231**	WT	c.839G>A	ER-, PgR-, HER2-	Basal	Metastasic adenocarcinoma
**MDA-MB-436**	c.5396+1G>A	c.? *	ER-, PgR-, HER2-	Basal	Adenocarcinoma
**MDA-MB-468**	Deletion exon 12	c.818G>A	ER-, PgR-, HER2-	Basal	Metastasic Adenocarcinoma

WT, wild-type; ER, estrogen receptor; PgR, progesterone receptor, HER2, human epidermal growth factor receptor 2; *Breast Cancer Cell line with controversial TP53 status (http://p53.free.fr/index.html; http://cancer.sanger.ac.uk/cosmic)
